# Editorial: Women in mucosal immunity

**DOI:** 10.3389/fimmu.2023.1282709

**Published:** 2023-10-03

**Authors:** Tanima Bose, Oscar Medina-Contreras, Carmen Fernandez, Susetta Finotto

**Affiliations:** ^1^ Biomedical Center, Ludwig-Maximilians-Universität (LMU), Munich, Germany; ^2^ Epidemiology, Endocrinology & Nutrition Research Unit, Mexico Children’s Hospital, Mexico City, Mexico; ^3^ Department of Molecular Biosciences, The Wenner-Gren Institute (MBW) Stockholm University, Stockholm, Sweden; ^4^ Department of Molecular Pneumology, Friedrich-Alexander-University (FAU) Erlangen-Nürnberg, Universitätsklinikum Erlangen, Erlangen, Germany

**Keywords:** environment, microbiome, fibrosis, SLE, IgGFc-binding protein, vaccination

Environmental triggers may profoundly affect host immune responses thereby causing structural alterations and tissue damage. However, the mechanisms by which persistent environmental triggers at local mucosal interfaces deregulate immunity are poorly understood.

In this Research Topic of the journal, five manuscripts address this important question.

Steele et al. describe how inflammatory processes at different organs result in increased extracellular matrix deposition and finally fibrosis (a leading cause of death in the world). They highlight that fibrosis is an unresolved chronic inflammatory process caused by environmental triggers, like bleomycin and carbon tetrachloride (CCl 4), in which members of the TNF superfamily play a pivotal role. In fact, TNFSF members appear to drive tissue fibrosis in various systems including heart, skin, gastrointestinal tract, kidney and liver. Whether in isolation or in combination with other anti-TNFSF member or treatment, targeting this superfamily remains key to improve efficacy and selectivity of currently available therapies for fibrosis.

The gastrointestinal tract has a large surface and is the first barrier against environmental triggers, such as food antigens or microorganisms. Thus, the local mucosa is a key player in providing host defense using innate and adaptive immune mechanisms. This is particularly relevant for the commensal microbiota which may cause local and systemic inflammation upon translocation into the intestinal wall. In this Research Topic, Gorman et al. identify the mucin MUC2 and the mucus associated protein FCGBP (IgGFc-binding protein), both major components of the colonic mucus, as crucial regulators of host defense and wound healing. This is due to a spatially coordinated expression and interaction of both proteins that results in enhanced epithelial barrier function. These results highlight a key role of glycans in the gut, first for the microbiota as a source of energy and nutrients and for the colonization of the gut; and second for the host as an important signaling pathway to regulate the immune system and host metabolism. Furthermore, the discovery of novel regulatory mechanisms on the composition, abundance, and interactions of glycans in the gut mucus can lead to novel therapeutic agents for intestinal inflammatory disorders.

An intact epithelial barrier function is tightly linked to immune homeostasis. The first microbial colonization occurs during birth when microbes from the birth canal and vagina are passed to the baby. By contrast, babies born by cesarean delivery are not exposed to this first microbial colonization which may result in an increased risk for the development of eczema, asthma, and celiac disease later in life. After birth, the microbiome is affected by various factors including breast feeding and nutrition. Breast milk itself has been shown to be a rich source of microbes and molecules with anti-inflammatory properties. Once a solid diet is introduced, the microbial species that populate the gut change significantly. Strains such as *Bacteroides* and *Firmicutes* help with the digestion of more complex carbohydrates and the production of vitamins. The gut microbiota is also involved in complex interactions with immune cells in the small intestine and the colon. Therapeutic strategies that target the microbiota — including dietary interventions, probiotics, short-chain fatty acids and faecal microbial transplantation — seem promising for immune dysregulation. However, disturbances of the gut microbiota have emerged as critical regulatory in allergies, and autoimmune disease and cancer. Systemic lupus erythematosus (SLE), an autoimmune diseases, is characterized by activation of the immune system with production of a large number of autoantibodies against nuclear components (**Figure 1**). The relationship between gut microbiome changes and metabolic alterations could help to explain the mechanisms by which gut bacteria control the pathogenesis of SLE. In this Research Topic, Ling et al. analyzed the gut microbiota and vagina microbiota and their function in lupus. Although the environmental trigger is not yet clear, the authors describe that SLE patients had fecal and vaginal dysbiosis, dysbiosis in vagina was more obvious than that in feces. These and future studies will provide us with novel opportunities to develop effective and precise diagnostic and therapeutic strategies and to explore potential microbiota-based treatments for patients with lupus.

**Figure 1:**
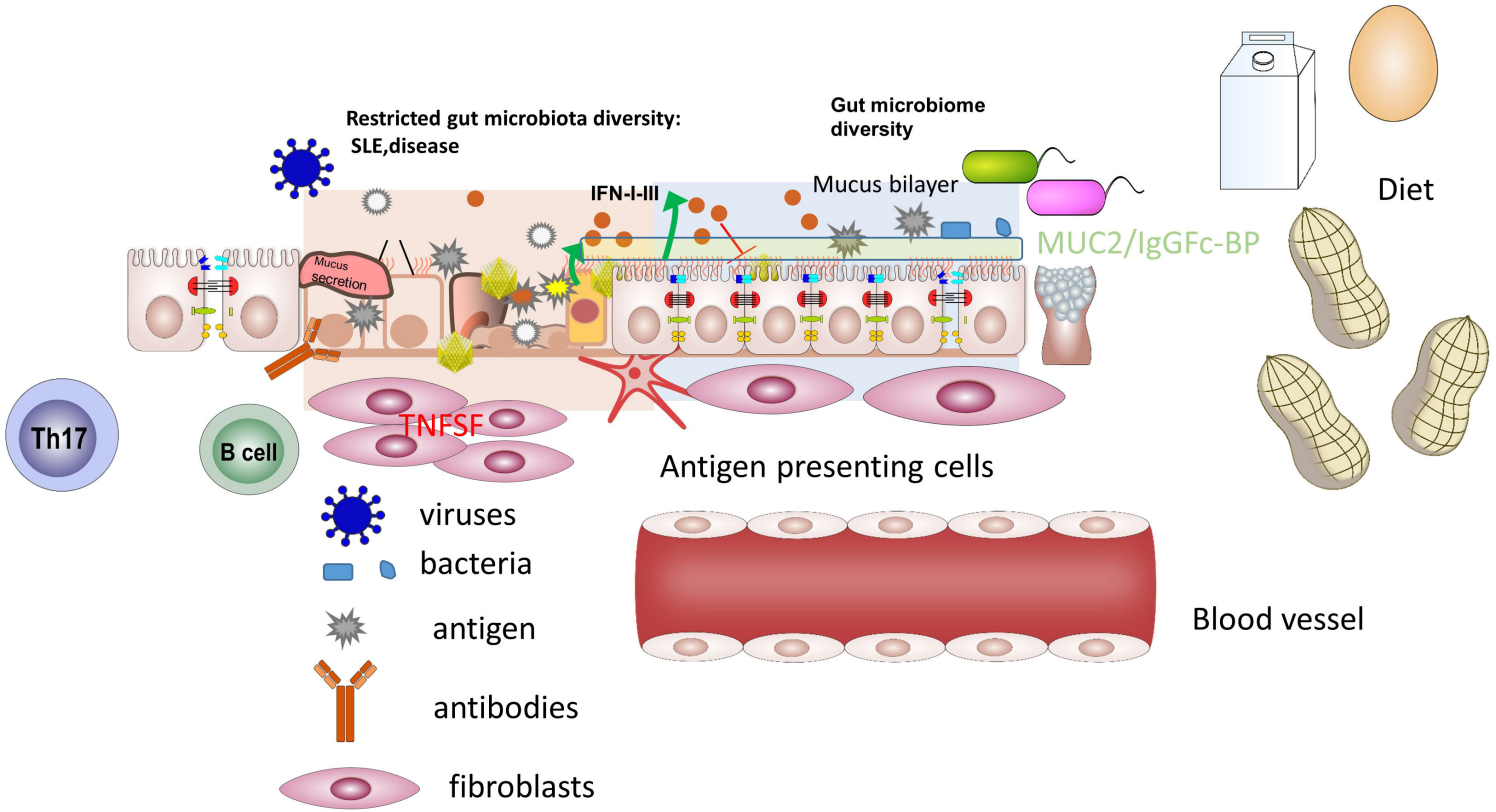
Environmental triggers in inflammation: clues to prevention and therapy of immune-mediated diseases. A. Pathologic deviations at the mucosal interfaces. B. Local protective components.

Responses induced locally in the respiratory tract are more efficient than responses induced systemically. Intranasal and oral delivery of antigens induces predominantly secretory IgA specific antibodies in the respiratory mucosa and specific antibodies in the serum. In contrast, systemic administration of the same antigens induces high antibody levels in serum with very low antibody levels in the respiratory tract. Thus, mucosal vaccination strategies may protect more efficiently against respiratory viral or bacterial pathogens by inducing immunity at the sites of infection. This concept could be also relevant for vaccination against SARS-CoV-2 which is investigated in the work presented by Braun et al. The authors propose that mucosal vaccination strategies may protect better against viral infection by inducing immunity at the sites of infection. Importantly, this approach may block viral transmission more effectively and contribute to inhibiting the evolution of new variants of concern (VOCs). This study was performed in hamsters where the authors compared intranasal, oral and systemic routes of vaccination. They showed that IgA and IgG responses induced by intranasal and oral vaccination are more efficient than the immune responses induced by other routes of vaccination and protected the animals upon challenge with a SARS-CoV-2 variant. The authors proposed that heterologous vaccination *via* mucosal routes may be advantageous for second-generation SARS-CoV-2 vaccines.

In the end, the paper by Petkov et al. addresses the local effects of an anti-retroviral drug on the skin mucosal system. This paper is important in delineating the mechanism by which local administration of drugs affects diseases. The authors demonstrated the transcriptomic effects of multiple drug doses and analyzed the subsequent functional modulation of mitochondrial stress, inflammation, and cell proliferation. Interestingly, the number of modulated genes increased exponentially with the number of doses of either oral tenofovir (TFV), disoproxil fumarate (FTC-TDF), or tenofovir alafenamide (FTC-TAF). This paper provided insights into the drug-modulatory effects of HIV drugs on the mucosal immune system on the skin and provides new ideas on how to investigate the dosing and immune-modulatory effects of anti-retroviral drugs. This paper gives a way to investigate further the dosing and safety criteria and immune-modulatory effects of these anti-retroviral drugs. This paper, though, does not invoke any new mechanistic action of the drug, but it entails the undesired effects of anti-retroviral drugs on the skin. Hence, this information will be helpful in the future while designing or approving these kinds of drugs.

The aim of this Research Topic was to identify environmental triggers that induce immune dysregulations in the host. The articles have identified several key mechanisms how environmental factors may affect host immunity and diseases. They have provided new insights into the pathogenesis of chronic inflammatory disorders and suggest new avenues for therapeutic concepts.

## Author contributions

TB: Data curation, Formal Analysis, Writing – original draft, Writing – review & editing. OM-C: Data curation, Formal Analysis, Writing – original draft, Writing – review & editing. CF: Data curation, Formal Analysis, Writing – original draft, Writing – review & editing. SF: Data curation, Formal Analysis, Writing – original draft, Writing – review & editing, Conceptualization, Project administration, Software, Supervision, Visualization.

